# Ventilator-associated pneumonia among intensive care nurses: a multicenter cross-sectional study on knowledge and preventive measures in intensive care units of Mogadishu, Somalia

**DOI:** 10.3389/fepid.2026.1770279

**Published:** 2026-06-05

**Authors:** Abdukadir Mohamed Hassan, Abdiwali Mohamed Hussein, Fathi yasin Yusuf, Abdishakur Mohamud hassan, Amal Abdullahi Abdi

**Affiliations:** 1 Department of ICU, Dr. Sumait Hospital, SIMAD University, Mogadishu, Somalia; 2 Department of Internal Medicine, Dr. Sumait Hospital, SIMAD University, Mogadishu, Somalia

**Keywords:** attitude, ICU nurses, knowledge, practice, ventilator-associated pneumonia

## Abstract

**Background:**

Ventilator-associated pneumonia (VAP) remains a major healthcare-associated infection in intensive care units (ICUs), and its prevention largely depends on nurses’ knowledge, attitudes, and adherence to recommended practices. Assessing these factors is essential for improving patient safety and the quality of ICU care. This study assessed the knowledge, attitudes, and preventive practices regarding VAP among ICU nurses in Mogadishu, Somalia.

**Methods:**

A multicenter descriptive cross-sectional study was conducted among 122 ICU nurses from five purposively selected hospitals in Mogadishu. Data were collected using a structured, validated self-administered questionnaire assessing socio-demographic characteristics, VAP-related knowledge, attitudes, and preventive practices. Descriptive statistics and Chi-square tests were performed using SPSS version 23, with statistical significance set at *p* < 0.05.

**Results:**

Most participants were aged 20–30 years (54.1%), male (52.5%), held a bachelor's degree (69.7%), and had received VAP training (73.8%). Overall, 85.2% of nurses demonstrated moderate knowledge, while 14.8% had good knowledge. Preventive measures such as hand hygiene (95.1%), sterile glove use (87.7%), and head-of-bed elevation (78.7%) were widely recognized. However, attitudes were predominantly poor, with 73.8% scoring below 50%, despite high recognition of oral care importance (83.6%). Preventive practices were also suboptimal, with 67.2% demonstrating poor practice levels. Although hand hygiene (73.8%) and glove use (69.7%) were commonly performed, only 50.8% consistently maintained the recommended semi-recumbent position. Significant associations were observed between socio-demographic characteristics and knowledge, attitudes, and practices (*p* < 0.001).

**Conclusion:**

ICU nurses demonstrated moderate knowledge but poor attitudes and preventive practices regarding VAP prevention. Structured training, standardized protocols, and institutional support are needed to improve compliance and enhance patient safety.

## Contributions to the literature

- Provides the first multicenter assessment of ICU nurses’ knowledge, attitudes, and preventive practices regarding ventilator-associated pneumonia in Somalia.
- Highlights gaps between knowledge and consistent implementation of evidence-based VAP prevention measures in a resource-limited ICU context.
- Adds evidence from a fragile health-system setting to the broader public health literature on healthcare-associated infection prevention.
- Informs priorities for targeted training, standardized protocols, and institutional support to strengthen infection prevention and patient safety.

## Background

Ventilator-associated pneumonia (VAP) is a significant and potentially life-threatening infection that develops in patients undergoing mechanical ventilation in intensive care units (ICUs). As the most common ICU-acquired pneumonia, VAP is associated with increased mortality, prolonged hospital stays, and substantial financial burdens, making it a major concern for healthcare systems worldwide (1–3). The incidence of VAP varies globally, with rates ranging from 7 to 43 cases per 1,000 ventilator days, and mortality rates reported between 6.3% and 66.9%, depending on the region and patient population (1, 2, 4).

Risk factors for VAP include prolonged mechanical ventilation, advanced age, comorbidities, emergency intubation, and certain medical interventions, while the most frequently implicated pathogens are multidrug-resistant Gram-negative bacteria such as Acinetobacter baumannii, Pseudomonas aeruginosa, and Klebsiella pneumoniae (1, 2, 5–7).

Nurses play a pivotal role in the prevention of VAP due to their continuous presence at the bedside and their responsibility for implementing infection control measures (2). However, evidence suggests that knowledge gaps and inconsistent adherence to prevention protocols among nursing staff, particularly in resource-limited settings, contribute to the ongoing burden of VAP (2).

In Somalia, and specifically in Mogadishu, there is a lack of reliable data on VAP prevalence and the effectiveness of preventive practices, underscoring the urgent need for well-structured research to inform targeted interventions. Assessing the knowledge, attitudes, and practices of ICU nurses regarding VAP prevention is essential for identifying educational needs, guiding policy, and ultimately improving patient outcomes in these high-risk environments.

## Material and methods

### Study design and setting

A descriptive cross-sectional study was conducted to assess the knowledge, attitudes, and preventive practices regarding ventilator-associated pneumonia among intensive care unit nurses in Mogadishu, Somalia. This design was selected to measure the current level of knowledge, attitudes, and practices within a defined population at a single point in time and to identify factors associated with preventive practices.

Mogadishu has ten hospitals with operational intensive care units providing mechanical ventilation services. Five hospitals were purposively selected based on predefined criteria, including availability of functional ICU services, routine management of mechanically ventilated patients, employment of qualified ICU nurses, and accessibility for data collection. The selected hospitals were Mogadishu Somali-Turkish Training and Research Hospital, Somali-Sudanese Specialized Hospital, Dr. Sumait Hospital, Yardimeli Specialist Hospital, and Kalkaal Hospital. These hospitals are major major public and private referral institutions delivering critical care services at primary, secondary, and tertiary levels.

Data collection was conducted over a four-month period, from June to September 2025.

### Study population and sampling procedure

The study population consisted of ICU nurses working in the selected hospitals during the study period. A purposive sampling method was used to recruit participants. This approach ensured inclusion of nurses with direct clinical responsibility for mechanically ventilated patients and relevant experience in ventilator care and infection prevention.

A total of 163 ICU nurses met the eligibility criteria across the five hospitals. Of these, 122 nurses participated in the study, resulting in a response rate of 74.8%. The distribution of participants by hospital was as follows: Mogadishu Somali-Turkish Training and Research Hospital (71 of 100), Somali-Sudanese Specialized Hospital (20 of 26), Dr. Sumait Hospital (15 of 16), Yardimeli Specialist Hospital (11 of 11), and Kalkaal Hospital (5 of 10).

All eligible nurses present during the data collection period were invited to participate. Data collection was conducted across different working shifts to maximize coverage. Nurses who were absent due to leave, off-duty schedules, or workload constraints were not included.

### Inclusion and exclusion criteria

Inclusion criteria were nurses currently working in intensive care units, having at least one year of ICU clinical experience, and providing informed consent.

Exclusion criteria were nurses not directly involved in the care of mechanically ventilated patients and nurses temporarily assigned to ICU units.

### Sample size determination

The sample size was determined using Cochran's formula for a single population proportion, assuming a 95% confidence level, a 5% margin of error, and a proportion of 50% because no prior local estimate was available. Since the total number of eligible ICU nurses in the selected hospitals was finite (*N* = 163), a finite population correction was applied. The final required sample size was 122 participants.

### Data collection instrument

Data were collected using a structured, self-administered questionnaire developed from validated instruments used in previous studies (1, 8–10). Each questionnaire was accompanied by an information sheet and informed consent form. Participants were assigned unique identification codes to ensure anonymity.

The questionnaire consisted of 27 items, divided into four sections:

#### Demographic characteristics

This section collected information on participants’ age, sex, marital status, educational level, duration of ICU experience, prior training in ventilator-associated pneumonia prevention, and ICU department (adult, pediatric, or neonatal).

#### Knowledge of ICU nurses regarding VAP

This section contained ten multiple-choice and yes/no questions covering general knowledge of ventilator-associated pneumonia, endotracheal tube management, patient positioning, suctioning procedures, and preventive measures. Each correct response was scored as 1 and each incorrect response was scored as 0, resulting in a total possible knowledge score ranging from 0 to 10.

This section was assessed using five items measured on a five-point Likert scale, ranging from strongly disagree to strongly agree. These items evaluated participants’ perceptions of the importance of VAP prevention, motivation for training, and adherence to evidence-based preventive practices. Responses were coded as strongly disagree = 1, disagree = 2, neutral = 3, agree = 4, and strongly agree = 5, resulting in a total possible attitude score ranging from 5 to 25.

This section assessed actual nursing practices using twelve items derived from the Centers for Disease Control and Prevention (CDC) VAP prevention guidelines (11). Topics included hand hygiene, glove use during oral care, subglottic suctioning, head-of-bed elevation, and VAP-related education. Higher-frequency responses indicated better preventive practice, and the summed practice score was converted to a percentage of the maximum possible score for categorization.

The KAP instrument consisted of 10 knowledge items, 5 attitude items, and 12 practice items. Knowledge items were scored as correct = 1 and incorrect = 0. Attitude items were scored on a five-point Likert scale from 1 to 5, and practice items were scored in ascending order according to response frequency, with higher scores indicating better preventive practice. Although reverse-coded items were initially considered, all items were positively worded and therefore no reverse coding was required.

#### Validity and reliability

The questionnaire was reviewed by a panel of five experts one academic specializing in critical and medical care, three ICU nurse specialists, one ICU and anesthesia specialist, and one general practitioner with ICU experience to assess content validity and clarity.

Reliability was established using Cronbach’s alpha, which demonstrated acceptable internal consistency:

Knowledge: *α* = 0.76
Attitude: *α* = 0.77
Practice: *α* = 0.79

A pilot study was conducted among ten ICU nurses to evaluate clarity and feasibility. Minor modifications were made based on feedback, and pilot participants were excluded from the final sample.

To ensure data quality, pre-testing was conducted with a subset of ICU nurses (excluded from the final study). The principal investigator and a field supervisor reviewed all completed questionnaires daily for completeness, accuracy, and consistency. Any discrepancies or missing data were addressed before final data entry.

#### Data analysis

Data were entered, coded, and analyzed using IBM SPSS Statistics version 23. Descriptive statistics, including frequencies and percentages, were used to summarize socio-demographic characteristics and knowledge, attitude, and practice levels. Raw domain scores were converted into percentage scores, and participants were categorized as having poor (<50%), moderate (50%–75%), or good (>75%) knowledge, attitude, and practice levels. Chi-square tests were used to assess associations between socio-demographic variables and the categorized knowledge, attitude, and practice levels and result were reported as *χ*^2^ statistic, degrees of freedom (df), *p*-value. A *p*-value of less than 0.05 was considered statistically significant, and values displayed by SPSS as 0.000 were reported as *p* < 0.001.

Thresholds were adopted from previously validated studies (10). For each domain, the participant's raw score was divided by the maximum possible score and multiplied by 100 to obtain a percentage score. Knowledge scores ranged from 0 to 10, with scores <5 categorized as poor, 5–7 as moderate, and ≥8 as good. The same percentage-based approach was applied to the attitude and practice domains after summing their item scores.

## Results

### Socio-demographic characteristics of the participants

A total of 122 ICU nurses participated in this study from five major hospitals in Mogadishu, The majority of ICU nurses were young, with more than half aged 20–30 years (54.1%), and males slightly predominated (52.5%). Most participants held a bachelor's degree (69.7%) and had 1–5 years of ICU experience (65.6%), indicating a relatively early-career but professionally qualified workforce. A high proportion (73.8%) had received formal training on ventilator-associated pneumonia prevention. Most nurses were assigned to adult ICUs (65.6%), while fewer worked in neonatal and pediatric units. Slightly more than half of the participants were single (50.8%) (Table 1).

**Table 1:** Socio-demographic characteristics of ICU nurses (*n* = 122).

Variable	Category	Frequency (n)	Percentage (%)
Age (years)	20–30	66	54.1
	31–40	46	37.7
	>40	10	8.2
Gender	Male	64	52.5
	Female	58	47.5
Education level	Diploma in Nursing	14	11.5
	Bachelor's Degree	85	69.7
	Master's Degree	23	18.9
ICU experience	<1 year	17	13.9
	1–5 years	80	65.6
	6–10 years	20	16.4
	>10 years	5	4.1
VAP training	Yes	90	73.8
	No	32	26.2
Marital status	Single	62	50.8
	Married	56	45.9
	Divorced	4	3.3
ICU unit	Adult ICU	80	65.6
	Pediatric ICU	19	15.6
	Neonatal ICU	23	18.9

### Nurse's knowledge regarding ventilator-associated pneumonia

Most nurses demonstrated adequate knowledge of ventilator-associated pneumonia (VAP) prevention. Nearly all (97.5%) correctly identified the full form of VAP, and 86.9% knew the proper disposal of suction catheters. About three-quarters (75.4%) recognized heat and moisture exchangers as the preferred humidifier type. Most respondents were aware that respiratory and bedside equipment should be cleaned once per shift (85.2%) and that elevating the head of the bed to 30–45° prevents VAP (78.7%). The importance of semi-recumbent positioning (80.3%), sterile gloves during suctioning (87.7%), and hand hygiene (95.1%) was also widely acknowledged. Overfeeding and unplanned extubation were identified as risk factors by 77.9% and 73%, respectively.

Overall, 85.2% of nurses had moderate knowledge, and 14.8% had good knowledge, with none showing poor knowledge. The overall pattern indicates a moderate level of knowledge among the participating nurses (Table 2).

**Table 2:** Nurses’ knowledge regarding ventilator-associated pneumonia (*n* = 122).

Knowledge item	Yes *n* (%)	No *n* (%)
VAP stands for ventilator-associated pneumonia	119 (97.5)	3 (2.5)
Single-use suction catheter disposal	106 (86.9)	16 (13.1)
Heat and moisture exchangers are preferred humidifiers	92 (75.4)	30 (24.6)
Equipment cleaning once per shift with antiseptic	104 (85.2)	18 (14.8)
Head elevation (30–45°) prevents VAP	96 (78.7)	26 (21.3)
Semi-recumbent positioning reduces VAP risk	98 (80.3)	24 (19.7)
Sterile gloves required for oral/ETT suctioning	107 (87.7)	15 (12.3)
Hand hygiene before/after suctioning is necessary	116 (95.1)	6 (4.9)
Overfeeding increases aspiration risk	95 (77.9)	27 (22.1)
Unplanned extubation increases VAP risk	89 (73.0)	33 (27.0)

Overall knowledge level		
Category	Frequency	Percentage (%)
Good (75%–100%)	18	14.8
Moderate (50%–74%)	104	85.2
Poor (<50%)	0	0.0

### Attitude regarding oral care and prevention of ventilator-associated pneumonia

Majority of nurses (46.7%) strongly agreed and 36.9% agreed that oral care is essential for VAP prevention. Regarding the effectiveness of current oral care practices, 38.5% strongly agreed and 28.7% agreed, while 20.5% remained neutral and 12.3% disagreed. Interest in receiving additional training was high, with 82% expressing agreement or strong agreement. Most nurses reported frequent implementation of oral care protocols (57.4% strongly agreed and 16.4% agreed), and over half expressed confidence in educating colleagues (53.3% agreed and 20.5% strongly agreed).

Despite recognizing the importance of oral care and training needs, nurses’ overall attitudes toward oral care and VAP prevention were predominantly poor 73.8% (<50%) (Table 3).

**Table 3:** Nurses’ attitudes toward oral care and VAP prevention (*n* = 122).

Item	Strongly agree *n* (%)	Agree *n* (%)	Neutral *n* (%)	Disagree *n* (%)	Strongly disagree *n* (%)
Importance of oral care in VAP prevention	57 (46.7)	45 (36.9)	15 (12.3)	5 (4.1)	0 (0.0)
Effectiveness of current oral care practices	47 (38.5)	35 (28.7)	25 (20.5)	5 (4.1)	10 (8.2)
Interest in additional training	45 (36.9)	55 (45.1)	15 (12.3)	5 (4.1)	2 (1.6)
Implementation of oral care protocols	70 (57.4)	20 (16.4)	25 (20.5)	5 (4.1)	2 (1.6)
Confidence in educating colleagues	25 (20.5)	65 (53.3)	25 (20.5)	5 (4.1)	2 (1.6)

Overall attitude level		
Level	Frequency	Percentage (%)
Good (≥75%)	7	5.7
Moderate (50%–74%)	25	20.5
Poor (<50%)	90	73.8
Total	**122**	**100**.**0**

**Table 4:** Nurses’ preventive practices regarding ventilator-associated pneumonia (*n* = 122).

Practice Item	Category	n	%
Hand hygiene between patients	Always	90	73.8
	Frequently	20	16.4
	Sometimes	10	8.2
	Rarely	2	1.6
	Never	0	0.0
Use of gloves during oral care	Always	85	69.7
	Frequently	22	18.0
	Sometimes	10	8.2
	Rarely	5	4.1
Patient positioning during suctioning	Semi-recumbent	62	50.8
	Lateral	30	24.6
	Supine	20	16.4
	Prone	10	8.2
Oral suctioning frequency	Every 4 h	57	46.7
	Every 8–12 h	40	32.8
	As needed	10	8.2
	Not performed	15	12.3
Tooth brushing frequency	Every 8–12 h	67	54.9
	Every 4 h	30	24.6
	As needed	25	20.5
Mouthwash used	Chlorhexidine	67	54.9
	Saline/others	40	32.8
	Hydrogen peroxide	10	8.2
	None	5	4.1

Overall practice level		
Level	n	%
Good (≥75%)	15	12.3
Moderate (50%–74%)	25	20.5
Poor (<50%)	82	67.2

### Preventive practices regarding ventilator-associated pneumonia

Nurses reported adherence to key ventilator-associated pneumonia (VAP) preventive practices. Hand washing between patient contacts was consistently performed by 73.8% of nurses and frequently by 16.4%. The majority (69.7%) always used gloves during oral care, while 18.0% did so frequently. During suctioning, half (50.8%) maintained patients in a semi-recumbent position, followed by lateral (24.6%) and supine (16.4%) positions. Oral suctioning was performed every 4 h by 46.7% and every 8–12 h by 32.8% of nurses. Tooth brushing was most often conducted every 8–12 h (54.9%) or every 4 h (24.6%). Chlorhexidine was the most commonly used mouthwash solution (54.9%).

Although most adhered to basic measures such as hand hygiene and glove use, overall compliance with comprehensive VAP prevention protocols remained suboptimal 67.2% (<50%) (Table 4).

### Association between socio-demographic variables and categorized knowledge, attitude, and preventive practice levels (Chi-square test)

Chi-square analyses showed statistically significant associations between selected socio-demographic variables and categorized knowledge, attitude, and preventive practice levels, as presented in Table 5.

**Table 5:** Association between KAP and demographic variables (Chi-square test).

Outcome	Variable	*χ* ^2^	df	*p*-value
Knowledge	Age	69.455	2	<0.001
	Gender	23.300	1	<0.001
	Education level	90.888	2	<0.001
	Experience	85.824	3	<0.001
Attitude	Age	125.579	4	<0.001
	Gender	47.865	2	<0.001
	Education level	85.189	4	<0.001
	Experience	164.015	6	<0.001
Practice	Age	136.533	4	<0.001
	Gender	65.669	2	<0.001
	Education level	89.502	4	<0.001
	Experience	101.326	6	<0.001

## Discussion

This study assessed knowledge, attitudes, and preventive practices (KAP) regarding ventilator-associated pneumonia (VAP) among ICU nurses in five major hospitals in Mogadishu, Somalia.

Overall, 85.2% of nurses had moderate knowledge regarding VAP prevention, and the majority correctly identified essential preventive measures, including head-of-bed elevation and sterile suctioning. Knowledge levels showed significant associations with selected socio-demographic characteristics. Similar findings have been reported in Delhi and the NCR region, where 54% of nurses had adequate knowledge of VAP prevention, and experience and specialized training were associated with better performance (12).

Despite recognizing the importance of oral care, our study found that 73.8% of nurses exhibited poor overall attitudes (<50%), even though 83.6% acknowledged its importance and 82% expressed willingness to receive further training. This has also been reported in other studies with nurses had negative attitudes toward oral care despite recognizing its preventive value, although 82% of nurses were willing to receive further training, 29% felt inadequately prepared, and only 65% adhered to oral care guidelines (13, 14).

Similarly, systemic challenges—such as lack of patient cooperation, inadequate staffing, insufficient equipment, and absence of standardized protocols—have been identified as barriers to effective oral care (15).

High compliance with basic infection control practices such as hand hygiene and glove use has been observed in this study but overall adherence to comprehensive VAP preventive measures was suboptimal, with Semi-recumbent positioning, a key intervention for reducing aspiration risk, was consistently maintained by only 50.8% of nurses. In contrast these studies (16–18) highlighted suboptimal semi-recumbent positioning due to workload pressures and inadequate supervision, and inconsistent oral suctioning intervals resulting from staffing shortages and absence of standardized protocols.

These findings suggest that improving VAP prevention in Somali ICUs requires multifaceted interventions. Knowledge alone does not guarantee optimal practice; positive attitudes, sufficient resources, standardized protocols, and supportive institutional policies are equally critical. Continuous education, practical workshops, simulation-based training, and mentorship programs can enhance nurses’ skills, confidence, and adherence to best practices. Hospitals should implement clear oral care and VAP prevention protocols, ensure availability of necessary equipment, and conduct regular compliance audits to foster a culture of patient safety and evidence-based care.

### Study limitations

This study has some limitations. First, the cross-sectional design limits the ability to establish causal relationships between socio-demographic factors and nurses’ knowledge, attitudes, and preventive practices. Second, purposive sampling and inclusion of selected hospitals in Mogadishu may limit the generalizability of the findings to all ICU nurses in Somalia. Third, data were collected using a self-administered questionnaire, which may be subject to reporting bias. Finally, preventive practices were not confirmed through direct observation, and therefore may not fully reflect actual clinical practice. Despite these limitations, the study provides valuable baseline information to guide future training and policy interventions for improving VAP prevention in Somali ICUs.

## Conclusions

ICU nurses in Mogadishu exhibited moderate knowledge but poor attitudes and preventive practices regarding VAP prevention. Socio-demographic characteristics, including age, education, gender, ICU experience, and professional role, were significantly associated with categorized KAP outcomes. Addressing these gaps through structured training, institutional support, and resource provision is essential to enhance preventive practices, reduce VAP incidence, and improve patient outcomes in Somali ICUs.

## Data Availability

The original contributions presented in the study are included in the article/Supplementary Material, further inquiries can be directed to the corresponding author.
